# The role of the surgeon in treating patients with lung cancer. An updating article

**DOI:** 10.1590/1516-3180.2020.0763.16022021

**Published:** 2021-05-28

**Authors:** Paulo Manuel Pêgo-Fernandes, Fabio José Haddad, Carlos Jogi Imaeda, Marcel Sandrini

**Affiliations:** I MD, PhD. Full Professor, Thoracic Surgery Program, Instituto do Coração (InCor), Hospital das Clínicas (HC), Faculdade de Medicina da Universidade de São Paulo (FMUSP), São Paulo (SP), Brazil; Cardiothoracic Surgeon, Hospital Beneficência Portuguesa (BP), São Paulo (SP), Brazil.; II MD, PhD. Thoracic Surgeon, Hospital Beneficência Portuguesa (BP) and Hospital Sírio-Libanês, São Paulo (SP), Brazil.; III MD. Thoracic Surgeon, Hospital Beneficência Portuguesa (BP), São Paulo (SP), Brazil.; IV MD. Thoracic Surgeon, Hospital Beneficência Portuguesa (BP), São Paulo (SP), Brazil.

**Keywords:** Pulmonary surgical procedures, Thoracic surgical procedures, Pneumonectomy, Thoracic surgery, Lung neoplasms, Lung cancer, Lobectomy, Pneumectomy

## Abstract

Lung cancer is a type of neoplasia with one of the highest incidences worldwide and is the largest cause of mortality due to cancer in the world today. It is classified according to its histological and biological characteristics, which will determine its treatment and prognosis. Non-small cell lung cancer accounts for 85% of the cases, and these are the cases that surgeons mostly deal with. Small cell lung cancer accounts for the remaining 15%. Surgery is the main method for treating early stage lung cancer, and lobectomy is the preferred procedure for treating primary lung cancer, while sublobar resection is an alternative for patients with poor reserve or with very small tumors. Surgeons need to be trained to use the resources and techniques available for lung resection, including less invasive approaches such as video-assisted thoracoscopic surgery (VATS) and robotic-assisted thoracoscopic surgery (RATS), and need to be familiar with new oncological approaches, including curative, adjuvant or palliative treatments for patients with lung cancer.

## INTRODUCTION

Lung cancer is the type of neoplasia responsible for the greatest number of deaths in Brazil and worldwide. Estimates from 2018 showed that lung cancer was among the forms with highest incidence, with 2.1 million new cases worldwide.^1^

The survival rate is low because lung cancer is diagnosed late and symptoms in the initial stages of the disease go unnoticed or do not exist.^2^ However, recently, a variety of advances have been changing this panorama, especially due to prospective studies on screening for lung cancer by means of low-dose chest computed tomography.^3^^,^^4^

Surgeons need to be aware that these studies have shown reductions in mortality due to lung cancer through use of screening protocols in at-risk populations, thereby increasing the number of diagnoses made at initial stages, in asymptomatic individuals. The survival of patients with advanced disease has also improved significantly over recent years, especially through the introduction of targeted molecular therapies and immunotherapy.^5^

## MAIN SUBTYPES

Lung cancer is subdivided into two major groups according to its histological characteristics, biological behavior, treatment and prognosis: non-small cell lung cancer (NSCLC), which accounts for 85% of the cases of malignant lung neoplasia; and small cell lung cancer (SCLC), which accounts for the remaining 15%.

Most cases of NSCLC are diagnosed at an advanced stage. This means that the survival rates are low, and this is seen especially in emerging countries, where scarcity of resources have the consequence that the main treatments are unavailable because of the high costs of tests and medications.^6^ Among cases of NSCLC, there are two main types: adenocarcinoma and squamous (or epidermoid) carcinoma.^7^ Squamous carcinoma accounts for 30% of NSCLC and was one of the most common types until the middle of the 1980s. This was the type most linked to tobacco smoking.^8^

## INITIAL ASSESSMENT OF PATIENTS WITH LUNG CANCER

Patients should be assessed by means of a detailed history and physical examination. They should be actively questioned about any history of smoking (even if this was passive smoking), exposure to toxic agents, oncological antecedents, family history of cancer and any signs and symptoms such as weight loss without apparent cause, loss of appetite, coughing, coughing with blood, hoarseness, dyspnea or chest pain, among others.^1^^,^^9^^,^^10^

Unfortunately, for most patients, their diagnosis is only made when the disease causes symptoms, i.e. when it is at an advanced stage. At this time, curative treatment is often no longer possible. Approximately 70% of the patients diagnosed with lung cancer in Brazil present locally advanced or metastatic disease.^6^

The diagnostic method should be chosen bearing in mind the patient's clinical condition, comorbidities, risk relating to invasive procedures and possibility of making the diagnosis and staging in a single procedure, with the aim of avoiding unnecessary invasive tests.^8^ The greater the amount of material that can be obtained and the better the definition of the staging that can be achieved within a single method, the better it will be for the patient.^11^ Some studies have shown that patients with lung cancer have better survival when therapeutic decisions are made by a multidisciplinary team.^12^ This is our opinion as well.

In addition to thoracic and upper abdominal computed tomography, the patient's clinical staging should be complemented through positron emission tomography–computed tomography (PET-CT) and magnetic resonance imaging (MRI) of the brain.

The test that is indicated when patients present with an initial peripheral lesion in one of the lungs is CT-guided needle biopsy. This method presents a diagnostic yield of up to 90%.^13^ On the other hand, central lesions are better evaluated by means of video-bronchoscopy, which enables a diagnosis and surgical planning. The diagnostic yield of bronchoscopy for central lesions is around 85%, when five or more fragments from the lesion are obtained.^11^

In the case of lesions associated with enlarged or PET-positive mediastinal lymph nodes, the methods that are indicated may be echobronchoscopy (EBUS) and/or mediastinoscopy. In patients with suspected metastatic lesions, the metastatic site should be targeted for biopsy. If it is proved that the suspected metastatic site is from a primary lung cancer, the diagnosis and staging are provided at the same time.^13^

## STAGING

Lung cancer staging is directly related to the patient's prognosis. This staging is based on the combination of three characteristics: size of the primary tumor and its invasion of adjacent structures (T); presence and extent of affected lymph nodes (N); and presence or absence of metastatic disease (M). This is the TNM staging system.

The TNM system (Table 1) has the objective of unifying the overall language of staging, thereby facilitating communication within care and academic studies.^14^ It also aids in selecting patients as candidates for surgical resection, radiotherapy, systemic treatment (chemotherapy, targeted therapy and immunotherapy) or combinations of two or more of these types of treatments.^15^

**Table 1:** Lung cancer stage grouping (eighth edition)^14^

T/M	Subcategory	N0	N1	N2	N3
T1	T1a	1A1	IIB	IIIA	IIIB
	T1b	1A2	IIB	IIIA	IIIB
	T1c	1A3	IIB	IIIA	IIIB
T2	T2a	IB	IIB	IIIA	IIIB
	T2b	IIA	IIB	IIIA	IIIB
T3	T3	IIIB	IIIA	IIIB	IIIC
T4	T4	IIIA	IIIA	IIIB	IIIC
M1	M1a	IVA	IVA	IVA	IVA
	M1b	IVA	IVA	IVA	IVA
	M1c	IVB	IVB	IVB	IVB

Adapted from the article “The eighth edition TNM stage classification for lung cancer: What does it mean on main street?”.^14^

## SURGICAL TREATMENT

Surgical treatment for lung cancer was first reported in 1933, when Evarts Graham performed the first left-side monoblock pneumonectomy, to resect a pulmonary tumor.^16^ Removal of the entire lung was the preferred procedure over the next 20 years, but this approach presented high rates of complications, including respiratory insufficiency, and deaths.

Pulmonary lobectomy with lymphadenectomy then became the gold standard for treating lung cancer in its initial stages.^17^ It was popularized through the studies conducted by William Cahan, published in 1962.^16^

Subsequent studies have shown the feasibility of sublobar resection for treating lung cancer in its early stages. In 1995, a randomized prospective study on lobectomy versus sublobar resection among patients in clinical stage IA of lung cancer was published. Lobectomy presented better results regarding local control and survival beyond two years after surgery.^18^

Criticisms of this study were made more recently, under the allegation that it was conducted in the pre-PET-CT era using old tomographs with poor anatomical definition. A further criticism was that patients with tumors of diameter up to 3.0 cm had been included in that study. Nonetheless, lobectomy continued to be the standard procedure for treating NSCLC, accompanied by hilar and mediastinal lymphadenectomy. Patients who are candidates for pulmonary resection need to be carefully selected, through evaluation of the histology of the tumor and the anatomical extent of the disease, and whether the patient is in a clinical condition to tolerate the proposed procedure. Patients who might benefit from the procedure include those with NSCLC in stages I and II and a limited group of patients with disease in stage III.^19^ Patients with SCLC in stage I may also undergo lobectomy and hilar and mediastinal lymphadenectomy, after invasive staging of the mediastinum to confirm that they have disease staged as N0.

Sublobar resection in the initial stages of lung cancer is a surgical option for treating primary pulmonary adenocarcinoma, especially for lesions smaller than 2.0 cm.^20^ However, this approach is still based on data from non-randomized studies.

The literature on sublobar resection for treating lung cancer only includes patients with adenocarcinoma. Other histological types should not enter within the criteria discussed above because of lack of support from scientific studies.^21^

The surgical approaches most commonly used for sublobar resection comprise wedge resection and segmentectomy. The former consists of removal of a pulmonary tumor together with a surrounding margin of normal lung tissue and is not an anatomical resection. On the other hand, segmentectomy is an anatomical resection in which one or more pulmonary parenchymatous segments are included, with dissection of segmental bronchovascular elements, together with intraparenchymatous, hilar and mediastinal lymph nodes.^22^ The approach of sublobar resection is not considered oncologically appropriate if any of the lymph nodes (intralobar, hilar or mediastinal) are found to be compromised by cancer.

In a multicenter prospective randomized study by Altorki et al. on 697 patients, the results from sublobar resection and lobectomy to treat peripheral tumors smaller than 2 cm were compared. It was demonstrated that perioperative morbidity and mortality did not differ among patients with NSCLC clinically staged as T1aN0, between those who underwent lobar resection and those who underwent sublobar resection.^23^ The survival results from that study, which are expected to be released in 2021, are keenly awaited.

The extent of adequate margins for sublobar resection continues to be discussed. In one study, it was recommended that the margins needed to be at least volumetrically greater than the largest diameter of the primary tumor.^24^ In some other studies, it was suggested that tumor margins > 1 cm were associated with significantly lower recurrence rates, in comparison with margins < 1 cm, in cases of primary tumors up to 1.0 cm in diameter.^20^

One important point to be taken into consideration in this scenario is the histological subtype of the adenocarcinoma. In the most recent classification of the World Health Organization, five main types of adenocarcinoma were established, namely: lepidic, acinar, papillary, micropapillary and solid.^25^ The lepidic type corresponds to adenocarcinoma *in situ* (or noninvasive). The last two subtypes (micropapillary and solid) correlate with worse prognosis, according to some studies. For some authors, these are also associated with higher rates of local recurrence in patients who undergo sublobar resection, by means of a form of local spreading through air spaces (STAS).^26^

The pathological definition of STAS is the presence of adenocarcinoma cells or groups of cells that have detached and become distant from the primary tumor, in alveolar spaces. It is known that some tomographic images correlate with certain subtypes of pulmonary adenocarcinoma, especially between ground-glass lesions and lepidic (noninvasive) adenocarcinomas. The other types of lesion are invasive and are characterized as solid nodules in tomographic examinations.

The micropapillary and solid subtypes seem to be more linked to STAS. In this regard, some authors have published data showing higher rates of local recurrence among patients undergoing sublobar resection, even at the initial stages of the disease.

Thus, lobectomy continues to be the standard procedure for treating primary pulmonary carcinoma. Sublobar resection is an alternative for patients with poor reserve or with tumors at a very early stage, generally with an invasive component smaller than 1.0 cm.^27^

Some authors have suggested that patients presenting peripheral pulmonary nodules with a ground-glass component that does not disappear during the follow-up are highly likely to have adenocarcinoma. This presentation should draw the surgeon's attention. A purely ground-glass lesion may evolve to a so-called semi-solid lesion (i.e. one with an invasive component). When this occurs, it becomes clearer that this carcinoma is turning aggressive and should be biopsied or surgically excised. If ground-glass lesions are manifested after resection of the lung cancer, this may be suggestive of recurrence of the neoplasia, or asynchronous multifocal disease.^28^

Dissection of hilar and mediastinal lymph nodes has become mandatory within the surgical treatment of lung cancer, given that is very important for staging, prognosis and treatment strategy. Nowadays, the presence of N1 or N2 lymph nodes determines that the surgeon will refer the patient to an oncologist, for assessment of adjuvant treatment.

The guidelines of the European Society of Thoracic Surgeons (ESTS) recommend sampling or dissection of lymph nodes in all patients with lung cancer who are candidates for surgical resection. Systematic evaluation of lymph nodes has been shown to be more effective than selective evaluation of lymph nodes. To obtain benefits from staging, it is recommendable to perform complete resection of the lymph nodes and the surrounding tissue, if feasible.^29^

In cases in which computed tomography or PET-CT shows suspicious mediastinal lymph nodes, invasive mediastinal staging becomes mandatory, with the initial objective of ruling out false positive results from imaging. Equally important, in addition to confirming whether N2 lymph nodes are compromised by neoplasia, is to be sure that the case is not one of occult N3. It is now known that the presence of N3 is a contraindication for surgical treatment, given that micrometastatic disease is highly likely to already be present.^14^

The presence of metastases in lymph nodes (either hilar or mediastinal) is a strong marker for the existence of micrometastatic disease. In these circumstances, and especially in situations of N2, there is a need for adjuvant or neoadjuvant chemotherapy. This should be defined together with the oncologist.

The main role of lymphadenectomy is well portrayed in a prospective study in which surgeons were asked to place lymph nodes that had been removed during the surgery, in specimen containers that had previously been labeled with the number of the lymph node chain, in accordance with the map of the International Association for the Study of Lung Cancer (IASLC). This method was tested and compared with what was done routinely by each surgeon in that service. The survival curves from the study showed significant differences in survival in favor of patients who underwent lymphadenectomy using so-called “lymph node kits”.^30^ Another study with an impact on this topic was conducted using the Surveillance, Epidemiology and End Results (SEER) database. The authors reviewed more than 24,000 cases of patients operated due to lung cancer. Those with the largest numbers of lymph nodes evaluated had better long-term survival.^31^

In services in which detection of cancerous mediastinal lymph nodes before surgery leads to neoadjuvant treatment, invasive staging of the mediastinum is practiced routinely. According to the ESTS guidelines, this staging is indicated for all cases of clinical N2 through imaging, cases of clinical N1 through imaging, cases of central tumors and cases of peripheral tumors of major diameter greater than 3.0 cm.^23^

In our service, all of these cases are discussed in multidisciplinary meetings, but we tend to recommend neoadjuvant therapy in these cases, provided that the staging is single-station N2. This is our limit for considering surgery as part of the curative treatment for these patients. For those presenting multiple-station N2, we follow the treatment protocol of the PACIFIC study, with definitive chemoradiotherapy followed by consolidative immunotherapy.^32^

It needs to made clear that we recommend invasive staging of the mediastinum before starting the treatment, and after multidisciplinary discussion. We agree with the ESTS guidelines that state that an abnormal mediastinum (suspected on computed tomography or PET-CT) should be confirmed through cytological or histological tests (endosonographic methods with cytological analysis and cell blocking, or surgical methods). In patients with a normal mediastinum, invasive staging of the mediastinum is recommended because of the possibility of occult N2, except in cases of peripheral tumors smaller than 3.0 cm (cT1N0). In cases of central tumors with the suspicion of N1 and in peripheral tumors larger than 3.0 cm, we think that procedures to exclude pathological involvement of the lymph nodes should be performed via endosonography (EBUS and/or EUS), or via surgical methods (mediastinoscopy, videomediastinoscopy, videothoracoscopy or anterior mediastinotomy).^29^

This approach has the main aim of ruling out situations of multiple-station N2, extracapsular invasion and N3 disease. We believe that these conditions are contraindications for surgery.

In situations of N2 disease, several approaches have garnered support in the literature. In some services, surgery up-front combined with adjuvant chemotherapy is recommended (IALT, JBR.10, ANITA).^33^ In other services, neoadjuvant systemic treatment followed by surgery is indicated. In our department, our preference is for neoadjuvant treatment after multidisciplinary discussion of the cases.

Surgical techniques for pulmonary resections have evolved, to include less invasive approaches. Video-assisted thoracic surgery (VATS) has become the preferred procedure for patients with NSCLC in early stages.^34^^,^^35^ VATS presents advantages regarding postoperative recovery, with fewer short-term complications, without prejudice to the oncological results. It is also a less painful procedure, with shorter hospital length of stay and, consequently, lower hospital costs.^36^^–^^38^

A meta-analysis conducted in 2012 showed that lobectomy through VATS yielded better perioperative outcomes than did lobectomy via thoracotomy. For instance, perioperative morbidity was significantly lower when VATS was used, including less occurrence of complications (prolonged air escape, pneumonia and sepsis) and shorter hospitalization.^38^ Although surgery using VATS demonstrates benefits, most patients who would be eligible for this minimally invasive procedure are still not treated in this manner.^37^^,^^39^

More recently, although no data have yet been published, the VIOLET study was presented at a IASLC meeting. In this prospective randomized study on surgical treatment for early-stage lung cancer, conducted in the United Kingdom, VATS was compared with thoracotomy for lobectomy. Despite a few methodological criticisms, this study was well designed, and demonstrated that morbidity was lower when VATS was used, with the same oncological results as when open surgery was used.^40^

Even today, most pulmonary lobectomies are performed through thoracotomy, both in developed and developing countries. This is because of limitations on financial resources that cause difficulty in implementation of VATS.^35^ It is also due to lack of training in this technique in many centers, with a long learning curve for most surgeons.

In Brazil, the outcomes from VATS lobectomies have been favorable regarding the surgical technique, complication rates and survival, as shown in a recent study by Tsukazan et al.^41^ However, even though the VATS approach is well established, it is still not the method most used in Brazil, given the scarce availability of resources and regional difficulties imposed by funding, considering that this type of procedure involves use of surgical staplers designed especially for minimally invasive surgery.^42^

The proportion of lung cancer patients who receive surgical procedures with curative intent in Brazil remains small: approximately 25% of all the patients diagnosed. This is due to the influence of factors such as presence of advanced disease at the time of diagnosis, poor performance status among the patients, presence of comorbidities and socioeconomic factors.^43^^–^^45^

Robotic surgery is the most recently introduced minimally invasive technique for treating lung cancer. Robotic platforms provide magnified three-dimensional visualizations of the surgical field that allow surgeons to perform complex delicate operations by means of small incisions. The equipment consists of a computer console at which the surgeon is positioned during the surgical procedure, where he or she manipulates robotic arms by means of the handles installed on the console. The robotic instruments have various degrees of movement resembling those of the human wrist. The computer converts the movements of the surgeon's hands into the same movements of the instruments.

The first reports on robotic surgery were from 2002. Studies have shown that general thoracic procedures can be performed safely with robotic tools, thus enabling precise dissection in areas that are difficult to access.^46^

Studies comparing lobectomy via thoracotomy, VATS and robotics have demonstrated that minimally invasive techniques present better results relating to length of hospital stay, mortality and postoperative complications.^45^ Some reviews using databases in the United States have, however, questioned the efficacy of this methodology. In a study on 15,502 patients who underwent pulmonary resection, either via VATS or robotics, the duration of the operation was longer in the cases of robotics-assisted surgery and the costs were higher, in comparison with other techniques.^47^

Table 2 presents brief definitions of the different surgical techniques, with their benefits and limitations.

**Table 2:** Brief definitions of the surgical techniques with their benefits and limitations^15^

Surgical techniques			
Surgical options	Brief definition	Benefits	Limitations
Wedge resection: Typically done via video-assisted thoracoscopic surgery (VATS) or robotic-assisted thoracoscopic surgery (RATS).	Small nonanatomical wedge is removed. Smaller than segmentectomy.	Preferred in patients with poor pulmonary reserve. Fewer complications and shorter hospital stays.	Generally not used for tumors > 4 cm. Location of tumor may preclude use of this option. May be associated with higher rate of local recurrence.
Segmentectomy: May be done via VATS and RATS.	Segment of lung is removed but not an entire lobe.	Larger parenchymal margins, and yields greater number of lymph nodes.	Larger than wedge resection but less than lobe. If margins are not clear, lobectomy may be needed.
Lobectomy	Removal of an entire lobe of lung, together with regional lymph nodes.	Gold standard for non-small cell lung cancer. Removal of entire lobe.	Preserves pulmonary function.
Pneumonectomy	Removal of entire lung and its lymph nodes.	Option when cancer involves proximal vascular structures or bronchi.	Higher mortality rate. More complications.

Adapted from the article “Surgical Treatment of Lung Cancer”.^15^

## FUTURE PERSPECTIVES

Assured access to healthcare services, with detection of early-stage lung cancer, may completely change the perspectives for and the progression of the disease. Incorporation of new technologies for minimally invasive surgery (VATS/RATS) has become a reality, in particular through research on new robotic platforms. Nonetheless, even in this scenario, treating multiple synchronic lung cancers remains a challenge, and these cases deserve a multidisciplinary approach.

Technology is helping to improve the results from surgical treatment of lung cancer, through use of three-dimensional planning. This can be used both for locating and assessing pulmonary nodules and for reconstruction of the thoracic wall when its resection and reconstruction are necessary.^48^

Concerning systemic therapy, a multicenter prospective randomized study on targeted adjuvant therapy was published recently. In this, patients undergoing surgery and who presented EGFR mutations were randomized to receive either osimertinib or placebo. The patients who received the medication presented an advantage regarding disease-free survival.^49^

With regard to advanced disease, an article published in the New England Journal of Medicine in which the SEER (surveillance epidemiology and end results) database was analyzed in the United States showed that there was an increase in cancer-specific survival relating to NSCLC between the years 2013 and 2016, after the introduction of targeted therapies and immunotherapy for these patients.^5^ Moreover, the concept of oligometastatic disease has been established, and surgeons need to be aware of this. It is possible that surgeons will take on increasing participation in approaches to treating patients with oligometastatic disease. Surgery may have a role in consolidation of local disease after initial systemic therapy.^50^

## CONCLUSION

Surgery is the cornerstone of treatment of early-stage lung cancer and, in selected cases, of locally advanced disease. It has a role, albeit less frequently, in cases of oligometastatic disease.

For early-stage disease (I and II), the surgical approach continues to be lobectomy with hilar and mediastinal lymphadenectomy. Sublobar resection is reserved for patients whose physiological reserves are too poor for lobectomy, or for patients with very small tumors (or a small solid part of the tumor). Indications for lymphadenectomy remain valid: this serves for staging and also has an impact on survival, and for recommending adjuvant treatment.

For patients in stage IIIA, a multimodal approach should be discussed in a multidisciplinary setting. Invasive staging of the mediastinum preceding the definitive surgical treatment or treatment with concomitant chemoradiotherapy is recommended, followed by consolidative immunotherapy.

Surgeons have a role in treatments for patients with oligometastatic disease, both for resection of primary tumors and for staging of the mediastinum before consolidative treatment. They therefore need to be familiar with the new technologies and new oncological approaches, in every scenario (curative or palliative). This makes surgeons key specialists in multidisciplinary groups that discuss treatments for patients with lung cancer.
